# Prediction model for spinal cord injury in spinal tuberculosis patients using multiple machine learning algorithms: a multicentric study

**DOI:** 10.1038/s41598-024-56711-0

**Published:** 2024-04-02

**Authors:** Sitan Feng, Shujiang Wang, Chong Liu, Shaofeng Wu, Bin Zhang, Chunxian Lu, Chengqian Huang, Tianyou Chen, Chenxing Zhou, Jichong Zhu, Jiarui Chen, Jiang Xue, Wendi Wei, Xinli Zhan

**Affiliations:** 1 Department of Spine and Osteopathy Ward, The First Affiliated Hospital of Guangxi Medical University, Nanning, Guangxi People’s Republic of China; 2 Department of Outpatient, General Hospital of Eastern Theater Command, Nanjing, Jiangsu People’s Republic of China; 3 Department of Spine Ward, Bei Jing Ji Shui Tan Hospital Gui Zhou Hospital, Guiyang, Guizhou People’s Republic of China; 4 Department of Spine and Osteopathy Ward, Bai Se People’s Hospital, Baise, Guangxi People’s Republic of China

**Keywords:** Spinal tuberculosis, Spinal cord injury, Machine learning, Predictive model, Model interpretation, Model deployment, Machine learning, Predictive medicine, Computational biology and bioinformatics, Microbiology

## Abstract

Spinal cord injury (SCI) is a prevalent and serious complication among patients with spinal tuberculosis (STB) that can lead to motor and sensory impairment and potentially paraplegia. This research aims to identify factors associated with SCI in STB patients and to develop a clinically significant predictive model. Clinical data from STB patients at a single hospital were collected and divided into training and validation sets. Univariate analysis was employed to screen clinical indicators in the training set. Multiple machine learning (ML) algorithms were utilized to establish predictive models. Model performance was evaluated and compared using receiver operating characteristic (ROC) curves, area under the curve (AUC), calibration curve analysis, decision curve analysis (DCA), and precision-recall (PR) curves. The optimal model was determined, and a prospective cohort from two other hospitals served as a testing set to assess its accuracy. Model interpretation and variable importance ranking were conducted using the DALEX R package. The model was deployed on the web by using the Shiny app. Ten clinical characteristics were utilized for the model. The random forest (RF) model emerged as the optimal choice based on the AUC, PRs, calibration curve analysis, and DCA, achieving a test set AUC of 0.816. Additionally, MONO was identified as the primary predictor of SCI in STB patients through variable importance ranking. The RF predictive model provides an efficient and swift approach for predicting SCI in STB patients.

## Introduction

Tuberculosis (TB), a global public health emergency, remains a significant threat to human health, and has a high mortality rate among infectious diseases^1,2^. Spinal tuberculosis (STB), the most common form of extrapulmonary tuberculosis, accounts for 50–70% of osteoarticular tuberculosis cases^3^. STB occurs when Mycobacterium tuberculosis travels through the bloodstream to the spine^4^. The early clinical signs of spinal tuberculosis are atypical, and as the condition progresses, it results in the destruction of bone and the spinal cord, leading to spinal cord injury and kyphosis^5,6^. Spinal cord injury (SCI) is one of the most common and serious complications in STB patients, and cause motor and sensory dysfunction, and even paraplegia, significantly affecting the physical and mental health of individuals^7^. Despite substantial efforts in diagnosing and treating spinal tuberculosis^6^, the prevention of complications in STB patients, especially those with SCI, remains challenging due to the presence of drug-resistant bacteria and late-stage detection^8^. An effective and concise prediction of SCI in STB patients is essential for establishing appropriate treatment plans and helping family members make informed decisions. However, predicting SCI in STB patients is challenging due to the complexity and variability of the syndrome, which involves various risk factors.

Machine learning (ML) algorithms are becoming increasingly crucial in various scientific domains^9^. The ML model, a subset of artificial intelligence, found applications in fields such as medicine, pharmacy, biology, and others^10–13^. Recently, there has been growing interest in using ML algorithms to study STB. For instance, Shuo D et al. accurately distinguished STB and spinal metastases on the basis of deep learning algorithms^14^. Li Z et al. developed a diagnostic model of STB from CT images and spinal metastases using deep learning algorithms^15^. They also developed a diagnostic model for STB using CT image features and deep learning. Moreover, several risk factors associated with STB have been identified through ML algorithms^16–18^. While several ML models have been employed to predict STB and identify risk factors, there is a lack of predictive models for SCI relying on ML algorithms. Therefore, there is an urgent need to establish a predictive model that healthcare professionals can trust to effectively predict SCI in patients with STB.

Our research aimed to create a practical model for predicting SCI in STB patients. To achieve this goal, multiple machine learning algorithms were utilized to develop a predictive model based on clinical data from patients with STB across three different hospitals.

## Materials and methods

### Patients

A review and analysis of pertinent medical data from 373 patients with STB at the First Affiliated Hospital of Guangxi Medical University, spanning from June 2012 to June 2021 were conducted to construct and validate the prediction model. Additionally, data from 100 STB patients at Bai Se People’s Hospital, Bei Jing Ji Shui Tan Hospital Gui Zhou Hospital, from July 2021 to January 2023 were collected to form a prospective cohort to test the prediction model. The inclusion criteria were as follows: (1) Patients with a confirmed diagnosis of STB. (2) Patients with no history of SCI resulting from other diseases. (3) Patients with no history of hematological system diseases. (4) Patients with complete clinical information. The exclusion criteria included were as follows: (1) Post-operative pathological diagnosis that did not confirm STB. (2) Complications with other diseases leading to SCI. (3) Complications with tumors, hematological system diseases, or immune system disorders. (4) Availability of only fragmentary information. Ethical approval for this study was obtained from the Ethics Committee at the participating hospitals (Supplementary Materials).

### Data gathering

The data, which included clinical characteristics and results from laboratory examinations, were gathered from patients who were admitted for the first time. General information about the patients, such as their age, gender, body mass index (BMI), presence of diabetes and hypertension, American Spinal Injury Association (ASIA) grade, oswestry disability index (ODI) scores, Japanese Orthopedic Association (JOA) score, and visual analog scale (VAS) rating, were collected. ASIA, ODI, JOA, and VAS scores were evaluated by two experienced specialists. The laboratory parameters consisted of the white blood cell (WBC)count, neutrophil count (NEU), lymphocyte count (LYM), monocyte count (MONO), C-reactive protein (CRP), erythrocyte sedimentation rate (ESR), hemoglobin (HGB), platelet(PLT), albumin (ALB), total protein(TP), aspartate aminotransferase (AST), alanine transaminase (ALT), urea, serum creatinine (Scr), and uric acid (UA) levels.

### Prediction model development, validation, and testing

After exclusions, a total of 329 patients from the First Affiliated Hospital of Guangxi Medical University were included in a retrospective cohort to create and validate the predictive model. Additionally, 80 patients from two other hospitals were included in a prospective cohort to test the model. In each cohort, all patients with complicated SCI (ASIA: A, B, C, D) were categorized as the SCI group, while the rest were classified as the No-SCI group. To further assess the severity, ODI, JOA, and VAS score were compared between groups.

The detailed processes of model construction were as follows: Dataset Partitioning and Data Imbalance Assessment: 329 patients were randomly divided in the retrospective cohort into a training set (n = 246) and a validation set (n = 83). Additionally, a total of 80 patients were defined in the prospective cohort as the test set. The ratio of the SCI group (n = 141) to the NO-SCI group(n = 105) in the training set was 1.34:1, indicating the absence of data imbalance issues^19^. Screening of characteristic indicators: Clinical characteristics and laboratory parameters with significant statistical differences were identified (p < 0.05) between groups through univariate analysis conducted in the training set. Systematic Analysis of multiple machine learning classifiers: Using the selected indicators, ten supervised ML classifiers were employed to construct prediction models. These classifiers included decision tree (DT), random forest (RF), Xtreme Gradient Boosting (XGBoost), least absolute shrinkage and selection Operator (LASSO) regression, support vector machine (SVM), multilayer perceptron (MLP), light gradient boosting machine (LightGBM), K-nearest neighbor (KNN), logistic regression, and stacking ensemble learning. To enhance the models, we performed hyperparameter tuning using grid search for each classifier. Grid search, a hyperparameter tuning technique, systematically explores a predefined set of hyperparameter values to identify optimal combinations for machine learning models. This process entailed creating a grid of hyperparameter values, where each point represents a unique combination. Subsequently, the model was trained and evaluated for each combination, with recorded performance metrics.The model was then trained and evaluated for each combination of hyperparameters, and the performance metrics were recorded^20^. To account for variations in model performance due to random data splits, a fivefold cross-validation procedure was performed in the training cohort^21^. Model Performance Evaluation and Optimal Model Selection: Model performance was evaluated using the receiver operating characteristic (ROC) curves, area under the curve (AUC), and precision-recall (PR) curves for each model^22,23^. Additionally, calibration curve analysis and decision curve analysis (DCA) were conducted to assess the robustness and clinical applicability of each model^24,25^. Based on the AUC, calibration curve analysis, and DCA results, the optimal model was determined. Subsequently, the AUC result was calculated for the test set to assess the performance of the optimal model. Model Agnostic Language for Exploration and Explanation (DALEX) Package: The DALEX package was used to explain the optimal model, quantify the contribution of each indicator to the predictive model and rank the importance of each feature. Moreover, the SHapley Additive eXplanation (SHAP) method was employed for single-sample prediction and interpretation^26,27^. Finally, the model was deployed on the web by an R Shiny app.

### Statistical analysis

The data analysis was conducted using SPSS (IBM version 26.0) and R statistical software (version 4.2.2). For continuous variables with a normal distribution, the *t* test was utilized, and the results are presented as mean ± standard deviation (SD). Continuous variables with a non-normal distribution were assessed using the Mann–Whitney *U* test, and the results are displayed as the medians (percentiles). Categorical variables were examined using either the chi-square test or Fisher’s exact test, and the outcomes are expressed as numbers (percentages). We considered statistical significance at a p-value less than 0.05.

### Ethics statement

Approval has been attained for the studies involving human respondents by the Ethics Department of Guangxi Medical University’s First Affiliated Hospital, the Ethics Department of Beijing Ji Shui Tan Hospital Guizhou Hospital and the Ethics Department of Baise People's Hospital.

### Consent form

Informed consent was obtained from all patients icluded in this study and/or their legal guardians. Following the requirements of national legislation and institution, informed consent was obtained from all participants and/or their legal guardians. All experiments and methods were performed in accrodance with relevat named guidelines and regulations.

## Result

### Patient clinical characteristics and laboratory results

**Figure 1:**
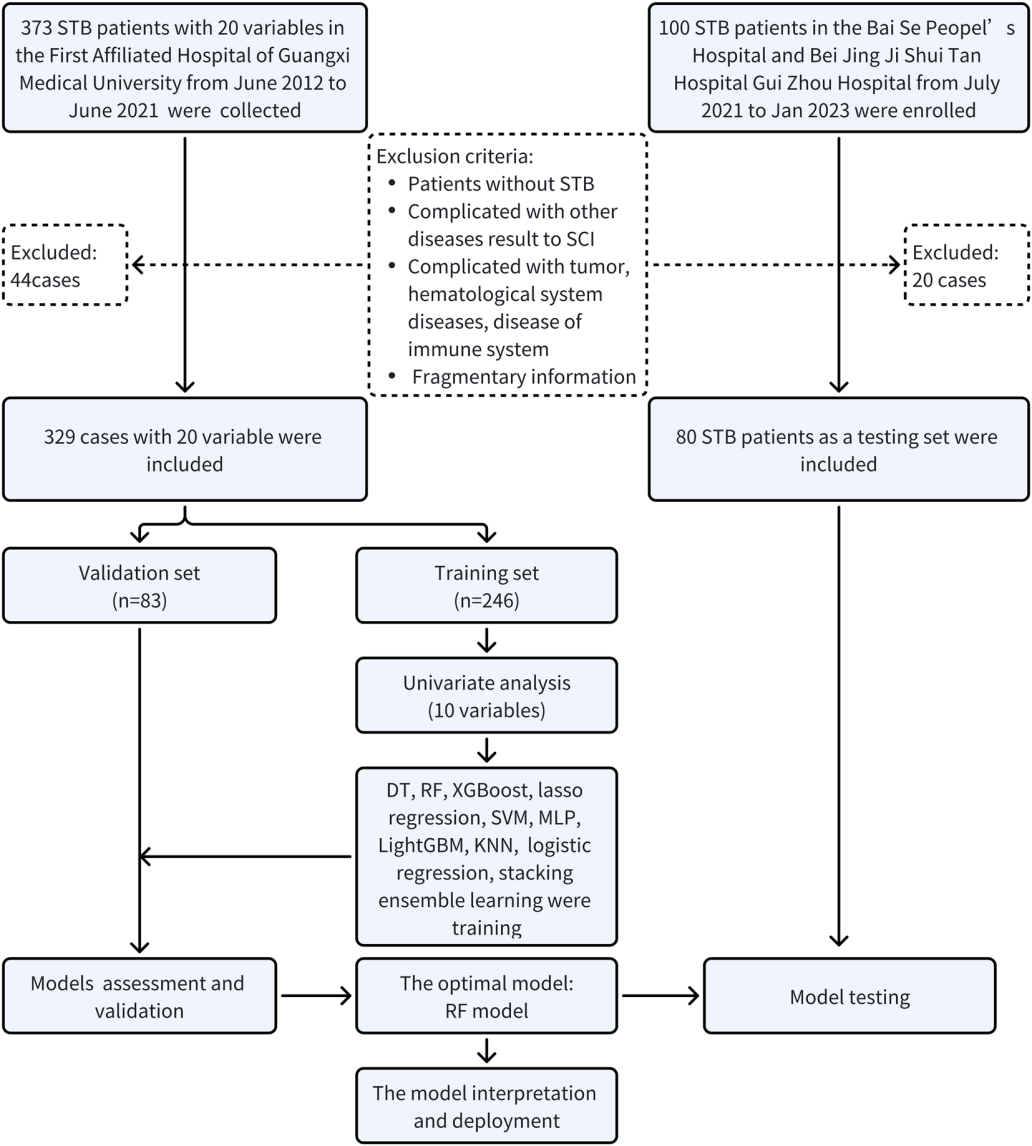
The flowchart of this study.

**Figure 2:**
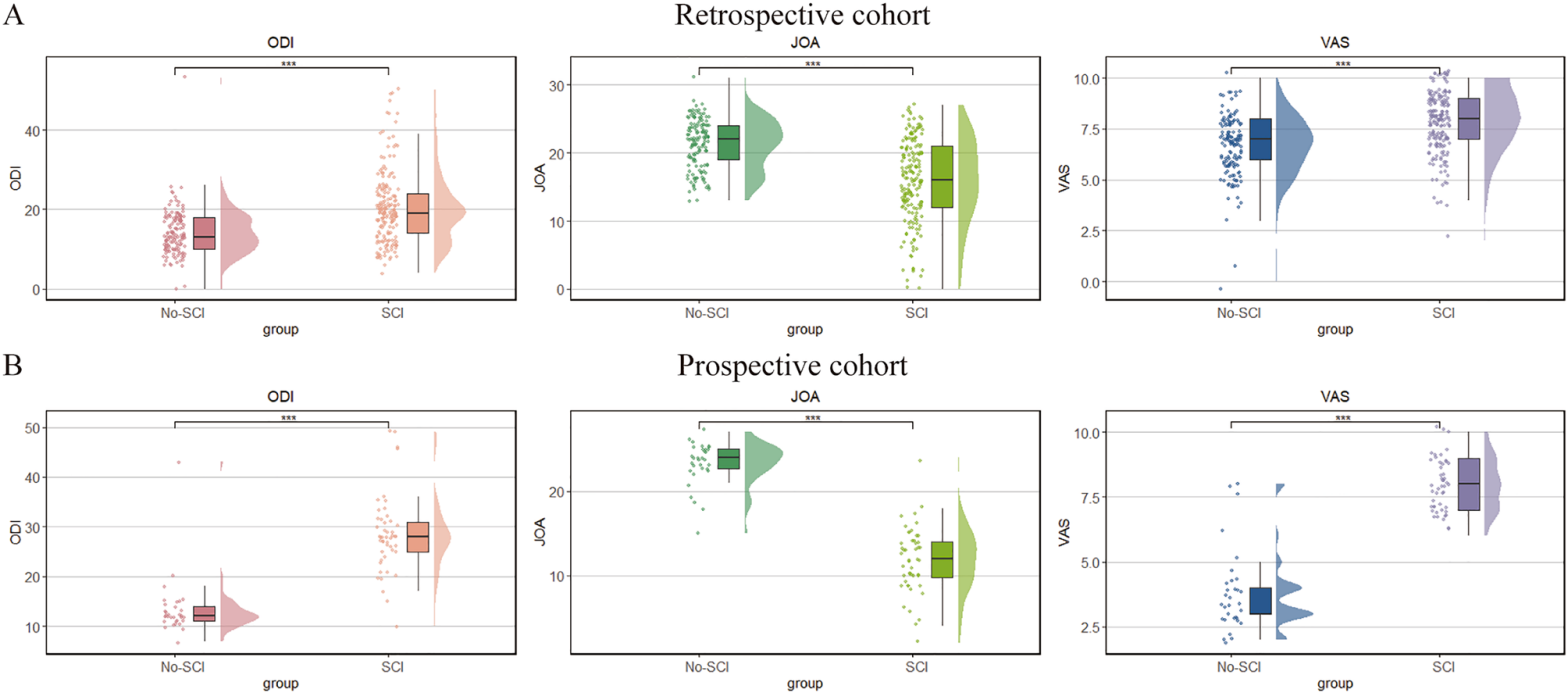
The severity of STB patients between two groups. (**A**) The differences in ODI, JOA, VAS scores between two groups in the retrospective cohort. (**B**) The differences in ODI, JOA, VAS scores between two groups in the prospective cohort. *ODI* oswestry disability index, *JOA* Japanese Orthopedic Association, *VAS* visual analog scale, *STB* spinal tuberculosis, *SCI* spinal cord injury. ***p-value < 0.001.

A total of 473 patients with STB were included from three medical institutions. After exclusions, we obtained a retrospective cohort consisting of 329 cases, including 246 cases in the training cohort and 83 cases in the validation cohort. Additionally, 80 patients in the testing cohort were included in the prospective cohort (Fig. 1). As demonstrated in Table 1, significant differences were found in age (p = 0.0003), complicated hypertension (p = 0.0006), CRP (p = 0.002), NEU (P = 0.0002), LYM (p = 0.0006), MONO (p = 0.0009), HGB (p = 0.0007), PLT (p = 0.0003), ESR (p = 0.0004), ALB (p = 0.0005) in the training set, while most indicators in validation and testing set had no difference (Supplementary Tables 1, 2). Notably, significant differences in MONO were observed in all the sets. Furthermore, as shown in Fig. 2, in the retrospective cohort and the prospective cohort, the ODI and VAS scores were significantly greater in the SCI group, while the JOA score was lower. These findings indicated that patients in the SCI group had a greater disease severity.

**Table 1:** Baseline characteristics of STB patients with and without SCI in training set.

Characteristics	No. (%)			p
	Total	SCI (n = 141)	No-SCI (n = 105)	
Age, years, median (IQR)	54 (37–66)	57 (41–67)	48 (34–61)	0.0003
BMI, Mean ± SD	20.9 ± 3.09	20.85 ± 2.85	20.89 ± 3.39	0.695
Sex, n (%)				0.079
Male	147 (59.8%)	83 (33.7%)	64 (26.1%)	
Female	99 (40.2%)	58 (23.6%)	41 (16.6%)	
Diabetes, n (%)				0.413
Yes	47 (19.1%)	24 (9.8%)	23 (9.3%)	
No	199 (80.9%)	117 (47.6%)	82 (33.3%)	
Hypertension, n (%)				0.0006
Yes	84 (34.1%)	61 (24.8%)	23 (9.3%)	
No	162 (65.9%)	80 (32.5%)	82 (33.4%)	
CRP, median (IQR)	14.3 (5.96–39.3)	21.9 (7.43–52.2)	10.8 (5.3–29.87)	0.002
WBC*10^9/L, median (IQR)	7.08 (5.8–8.34)	7.2 (6.04–8.66)	6.9 (5.56–7.96)	0.06
NEU*10^9/L, median (IQR)	4.53 (3.53–5.77)	4.73 (3.81–6.07)	4.08 (3.19–5.32)	0.0002
LYM*10^9/L, median (IQR)	1.46 (1.09–1.79)	1.38 (1.03–1.66)	1.62 (1.24–1.91)	0.0006
MONO*10^9/L, median (IQR)	0.61 (0.48–0.77)	0.63 (0.52–0.81)	0.58 (0.44–0.69)	0.0009
HGB g/L, median (IQR)	123 (111–133)	117 (106–131)	126 (117–135)	0.0007
PLT *10^9/L, median (IQR)	291 (247–351)	310 (261–371)	269 (237.6–309)	0.0003
ESR, median (IQR)	40 (23.2–60)	44 (30–67)	34 (17–55)	0.0004
ALB g/L, median (IQR)	38.6 (35.2–41.4)	37.7 (33.3–39.9)	39.3 (37.2–42)	0.0005
TP g/L, median (IQR)	71.3 (67–74.9)	71.3 (66.6–75.9)	71.3 (67.6–74)	0.919
AST u/L, median (IQR)	20 (17–26)	20 (17–26)	20 (17–27)	0.715
ALT u /L, median (IQR)	16 (12–23)	16 (12–22)	17 (12–24)	0.482
Ure u/L, median (IQR)	4.26 (3.35–5.21)	4.32 (3.36–5.44)	4.23 (3.34–5.13)	0.554
Scr u/L, median (IQR)	67 (56–77.8)	67 (55–77)	67 (57–79)	0.858
UA u/L, Mean ± SD	401 ± 166	398.16 ± 173.04	404.47 ± 157.01	0.586

### Feature screening

To enhance the models’ performance, univariate analysis was employed to examine the variables. As depicted in Table 1, significant differences in age, complicated hypertension, NEU, LYM, MONO, CRP, ESR, HGB, PLT, and ALB were identified between the SCI group and the No-SCI group in the training set. These 10 clinical features were subsequently utilized to construct the predictive model.

### Comprehensive analysis of multiple machine learning algorithms

**Figure 3:**
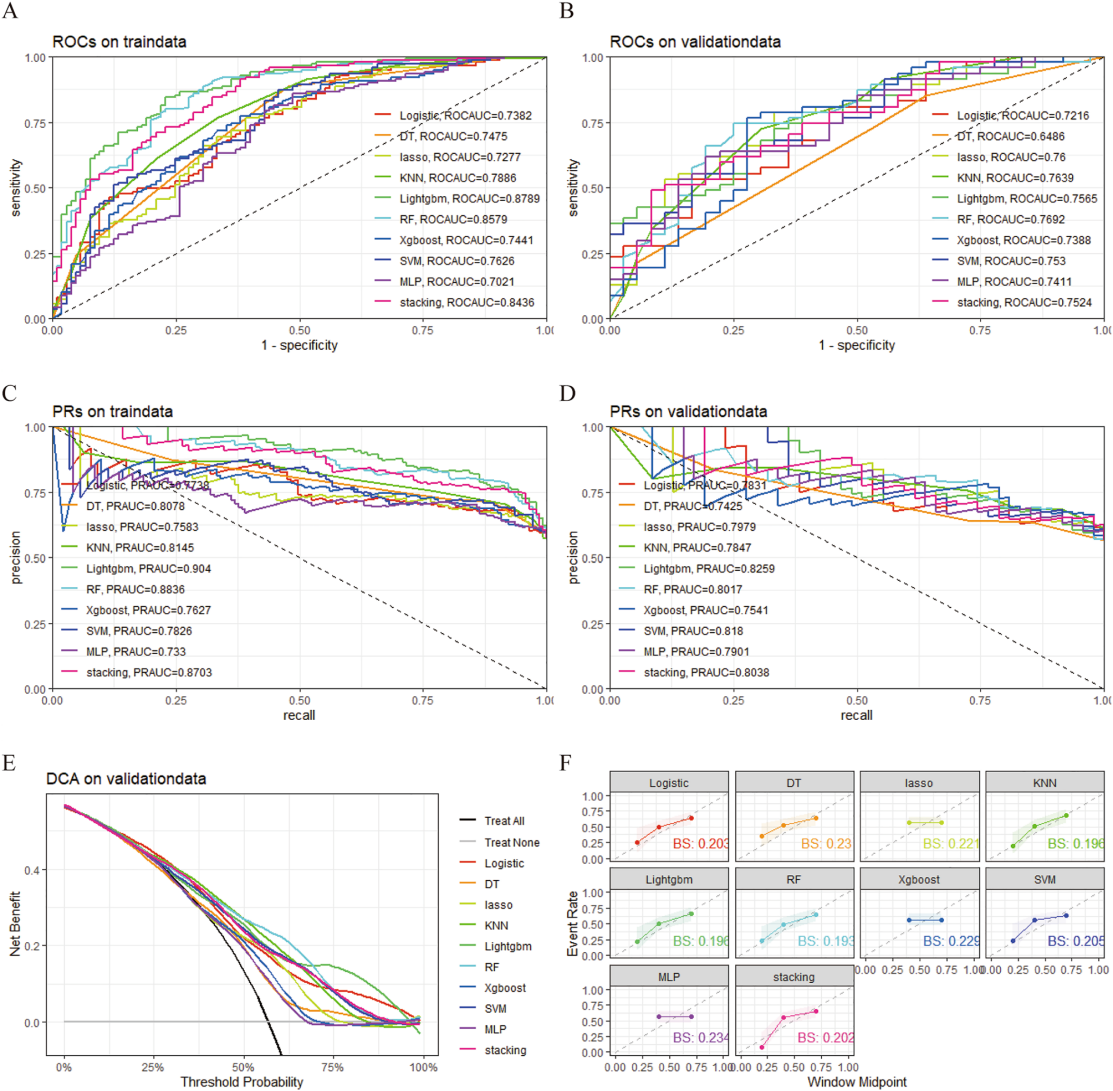
Comprehensive analysis of mutiple ML algorithms. (**A**) ROC and AUC value of ML models in training set. (**B**) ROC and AUC value of ML models in validation set. (**C**) PRs of ML models in training set. (**D**) PRs of ML models in validation set. (**E**) DCA of ML models in validation set. (**F**)The calibration curve of ML models in validation set. *ML* machine learning, *ROC* receiver operating characteristic curves, *AUC* area under the curve, *PRs* precision-recall curves, *DCA* decision curve analysis, *DT* decision tree, *RF* random forest, *XGBoost* Xtreme gradient boosting, *LASSO* least absolute shrinkage and selection operator, *SVM* support vector machine, *MLP* multilayer perceptron, *LightGBM* light gradient boosting machine, *KNN* K-nearest neighbor, *bs* brier score.

We trained ten different models, namely, DT, RF, XGBoost, LASSO regression, SVM, MLP, LightGBM, KNN, logistic regression, and stacking ensemble learning. Subsequently, the models were evaluated using AUC values. The results indicated that in the training cohort, LightGBM, RF, and the stacking ensemble learning model performed the best^22^, with RF achieving the highest AUC in the validation cohort (Fig. 3A and B). The results of the precision-recall curves demonstrated that in the training cohort, the LightGBM, RF, stacking ensemble learning, and KNN models outperformed the other models, while in the validation cohort, the LightGBM, SVM, stacking ensemble learning, and RF models outperformed the other models (Fig. 3C and D). Moreover, decision curve analysis (DCA) and calibration curve analysis were conducted to assess the clinical efficacy of each machine learning model. DCA revealed that RF, LightGBM, and KNN were better suited for clinical application (Fig. 3E). Consistently, RF, LightGBM, and KNN demonstrated higher accuracy according to the calibration curves (Fig. 3F). Based on these results, we conclude that RF can be considered as the best performing model.

### Optimal model establishment and assessment

**Figure 4:**
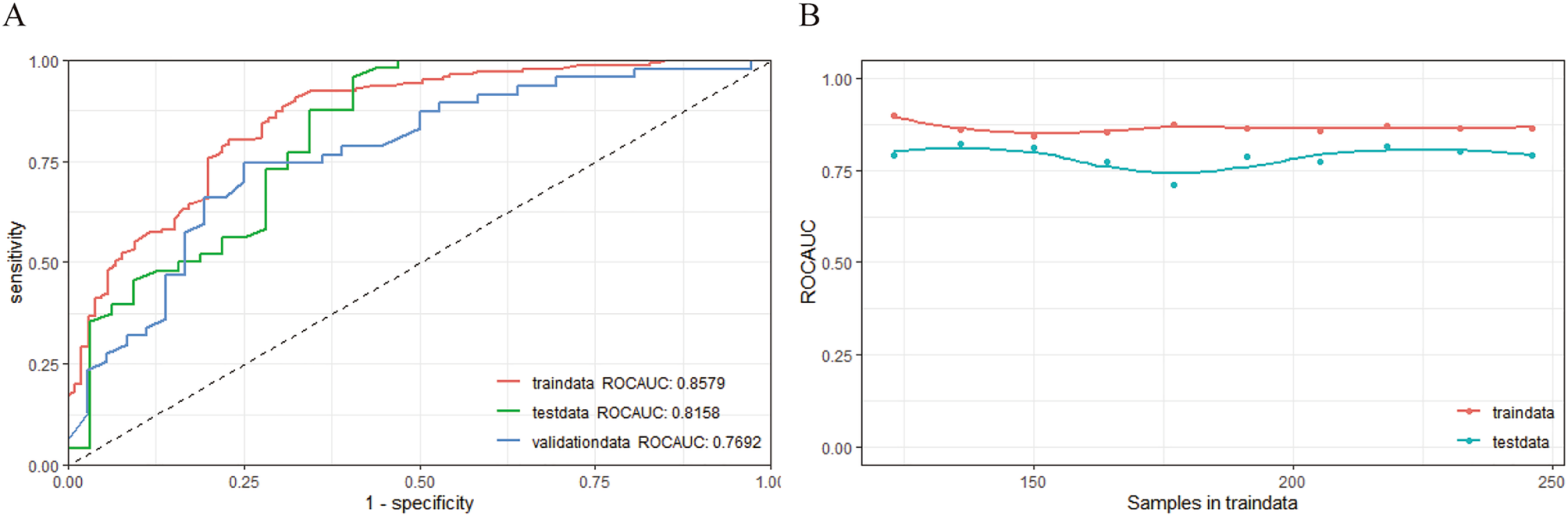
Random forest model assessment. (**A**) ROC and AUC value of random forest model in training, validation and testing set. (**B**) Learning curve. *ROC* receiver operating characteristic curves, *AUC* area under the curve, *RF* random forest.

The prediction model was constructed using the random forest (RF) algorithm. In Fig. 4A, the AUC values for the training set (AUC = 0.858), the validation set (AUC = 0.769), and the testing set (AUC = 0.816) are displayed. The model was considered successful since the AUC value in the testing set was higher than that in the validation set. Furthermore, the learning curve indicated a favorable fit and stability between the training and validation sets (Fig. 4B)^28^. In addition, based on our calibration procedures, we observed consistent performance of the RF model's probability outputs across different probability levels, with the calibration curve closely resembling the ideal 45-degree diagonal line (Fig. 3F). The results of statistical metrics indicated that the RF model's Brier score (0.193) reached reasonable levels, further confirming the effectiveness of our model's probability calibration. Therefore, the RF model is a useful approach for predicting SCI in patients with spinal tuberculosis (STB).

### Prediction model interpretation and deployment

**Figure 5:**
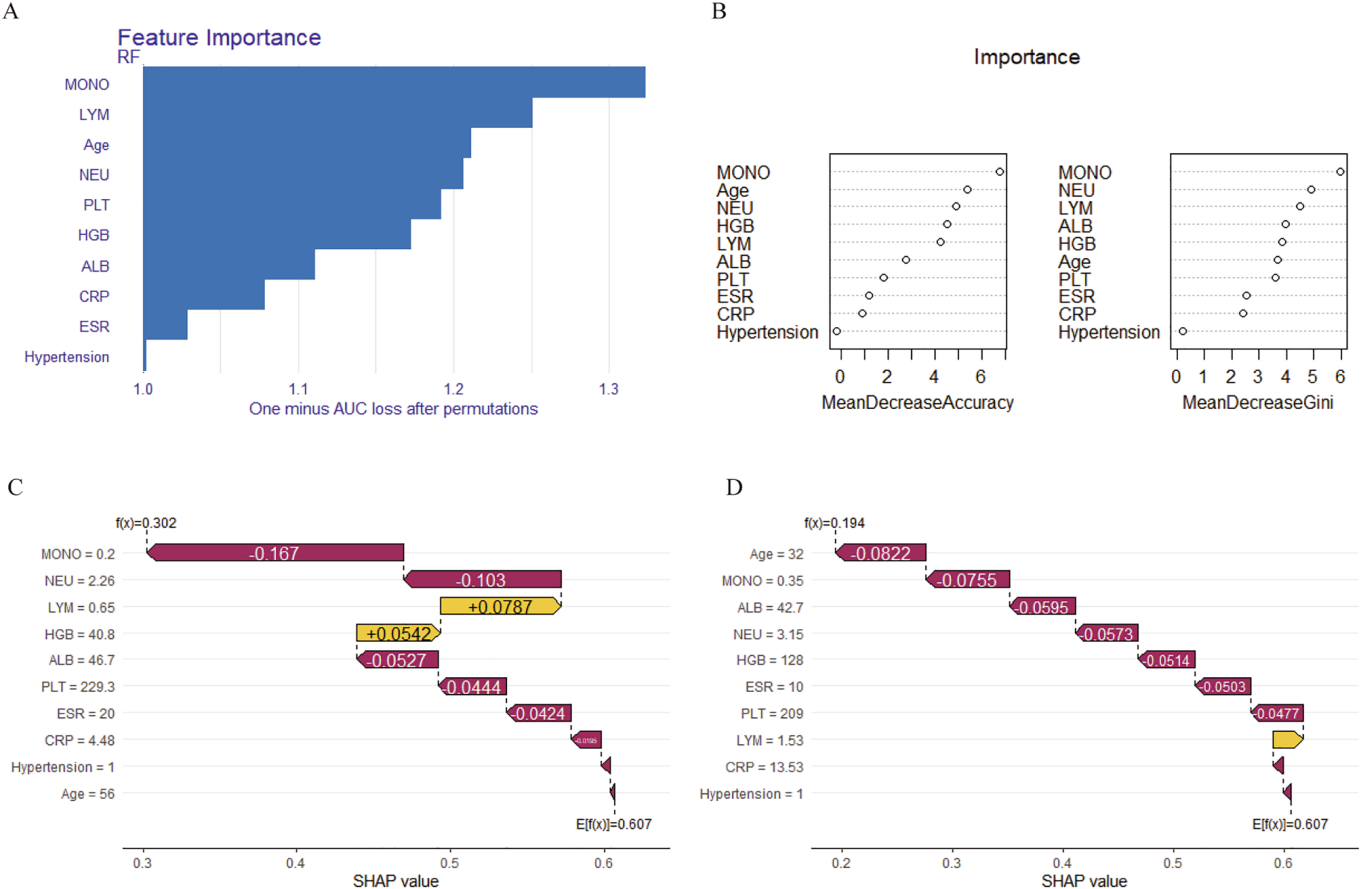
The model interpretation. (**A**,**B**) Feature importance ranking contribute to the model. (**C**) The model interpretation in one patient without SCI by SHAP. (**D**) The model interpretation in one patient with SCI by SHAP. *NEU* neutrophil count, *LYM* lymphocyte count, *MONO* monocyte count, *CRP* C-reactive protein, *ESR* erythrocyte sedimentation rate, *HGB* hemoglobin, *PLT* platelets, *ALB* albumin, Hypertension, combined hypertension.

**Figure 6:**
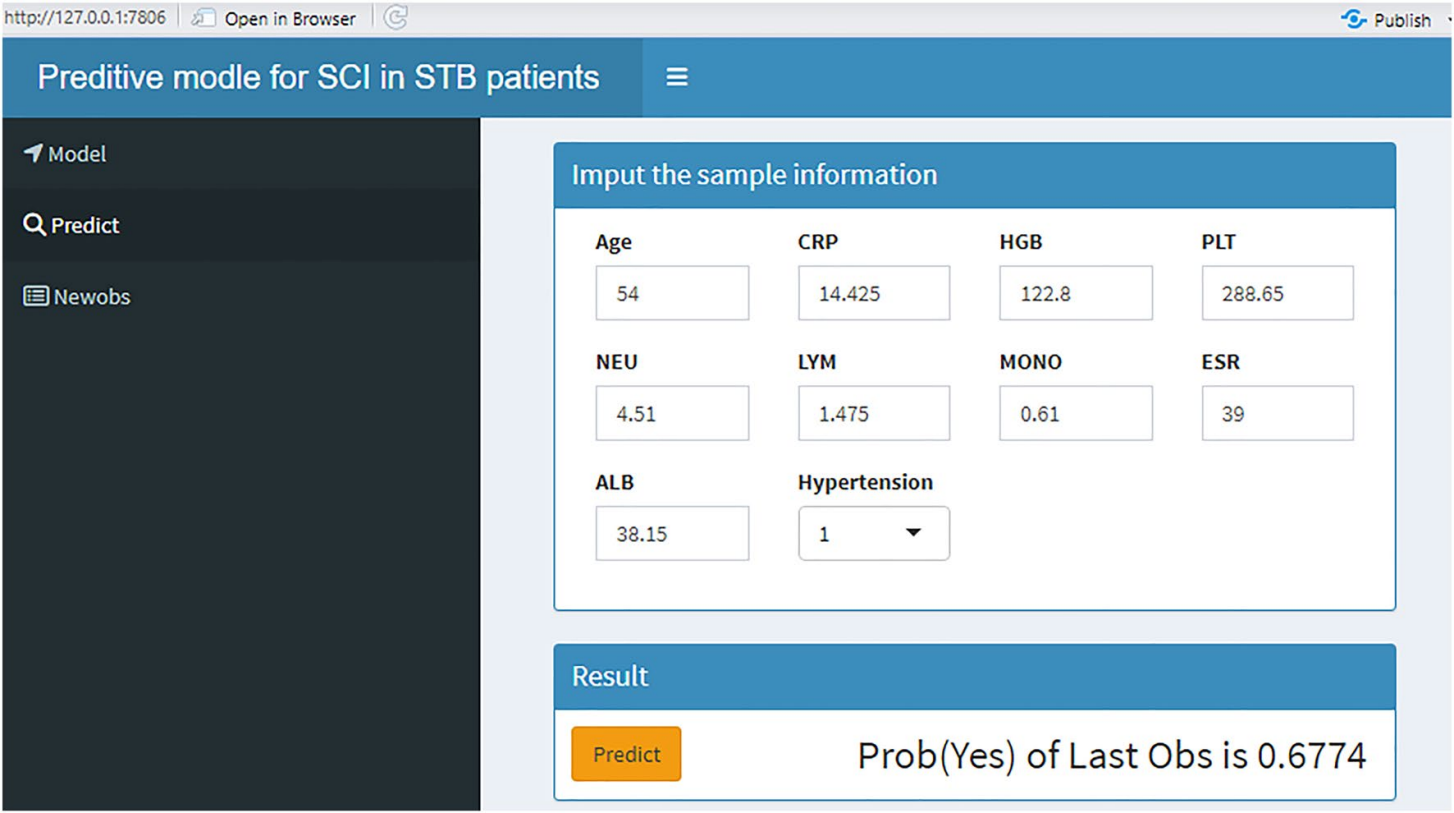
The model deployment. *NEU* neutrophil count, *LYM* lymphocyte count, *MONO* monocyte count, *CRP* C-reactive protein, *ESR* erythrocyte sedimentation rate, *HGB* hemoglobin, *PLT* platelets, *ALB* albumin, Hypertension, combined hypertension.

We employed the DALEX R package to elucidate how the selected parameters predict the progression of spinal tuberculosis and assess their importance in the model. In Fig. 5A and B, the rankings of importance of the ten features are presented, with MONO emerging as the most significant factor for SCI in STB patients. To enhance the interpretability of the model, two representative samples were provided using the SHAP model. One sample was from an STB patient without SCI (Fig. 5C), while the other belonged to the SCI group (Fig. 5D). Finally, Fig. 6 shows the predictive model constructed via the web (http://127.0.0.1:7806).

## Discussion

STB tends to affect younger individuals and is not uncommonly associated with SCI, which can lead to disability and even death^29,30^. The progression of STB is a major contributor to severe spinal complications and poses a significant challenge for achieving positive outcomes in STB patients. Therefore, timely identification of patients at risk of experiencing spinal cord injury and identification of key factors involved in disease progression are crucial in clinical practice. In this study, we identified important clinical and laboratory examination characteristics and employed a beneficial ML-based model to predict the occurrence of SCI in STB patients. We believe that ML-based models are valuable tools in clinical practice because they are noninvasive, rapid, and user-friendly, aiding in the prediction of spinal cord injury in STB patients.

SCI results from the progression of STB. Given the low early detection rate and poor prognosis of STB patients, there is a growing focus on identifying biomarkers for the occurrence and development of this disease^16,17,31,32^. Several clinical indicators related to the progression and prognosis of STB have been investigated. Immune cells, key players in immune function, play a crucial role in the progression of STB. For instance, Yao Y et al. identified two subphenotypes of spinal tuberculosis of varying severity using unsupervised machine learning and found significant differences in the infiltration levels of immune cells (lymphocytes, monocytes, neutrophils), which are related to SCI^33^. A multicenter study demonstrated that the monocyte-to-lymphocyte ratio (MLR) and neutrophil-to-lymphocyte ratio (NLR) were notably greater in patients with active tuberculosis than in those with latent tuberculosis^34^. Additionally, well-known indicators,such as ESR and CRP concentration, are commonly used to evaluate the degree of inflammation in patients with tuberculosis^35^ and are associated with the prognosis of STB^36,37^. Nutritional status is also a vital factor affecting the progression and prognosis of spinal tuberculosis patients, and research has shown that the serum ALB concentration is an important laboratory marker for predicting SCI and prognosis in STB patients^38^. In this study, we ultimately selected ten clinical parameters, including age, NEU, LYM, MONO, CRP, ESR, HGB, PLT, ALB, and complicated hypertension, to establish the predictive model. The results consistently indicated that patients in the SCI group were more likely to be older and to have complicated hypertension, NEU, MONO, CRP, ESR, and PLT than were those in the No-SCI group, while LYM, HGB, and ALB were more common in the No-SCI group.

Moreover, based on the importance ranking of the ten clinical factors, MONO was identified as the most significant predictor. Recent studies have suggested a close association between the dysregulation of monocytes and disease progression. A retrospective study demonstrated that a high monocyte-to-lymphocyte ratio (MLR) was closely linked to the severity and occurrence of SCI in patients with spinal tuberculosis^39^. Another recent study reported that elevated monocyte levels contributed to the progression of tuberculosis^40^. Furthermore, a single-center retrospective study revealed that a high monocyte-to-lymphocyte ratio was a risk factor for clinical progression in patients with pulmonary *Mycobacterium avium* complex disease^41^. Additionally, a growing body of research indicates that monocytes and macrophages play crucial roles in the prognosis of non-infectious diseases^34,42^. Interestingly, the results of the present study align with the findings of the aforementioned studies, revealing a markedly elevated level of monocytes in the SCI group. Therefore, monocytes may be a crucial factor in the progression of spinal tuberculosis and may be involved in the development of spinal cord injury in STB patients.

Monocytes, which originate from monocyte precursors in the bone marrow, are recruited to infection sites. These cells differentiate into macrophages when they respond to antigens to defend against infections^43^. These macrophages are part of the mononuclear phagocyte system and play crucial roles in defense mechanisms, tissue development, and maintaining the body’s balance^44^. The abilities of these bacteria to engulf foreign particles and kill bacteria, as well as produce inflammatory cytokines, are important for supporting adaptive immune responses. Monocyte-macrophages, acting as a “double-edged sword,” are essential for defending the body against pathogenic infections, but their hypersensitive reactions can lead to damage to normal tissues and organs during infections^45^. Additionally, they can play both protective and pathogenic roles in various diseases^46^, which depend on the surrounding environment that regulates their phenotype and function. There are two main subtypes of macrophages: (1) Classically activated M1 macrophages, which typically produce pro-inflammatory cytokines such as TNF-α, IL-1β, IL-12, and IL-23, promoting local inflammation and helping eliminate pathogens, virus-infected cells, and transformed cells. (2) M2 macrophages generally produce anti-inflammatory cytokines such as IL-10 and TGF-β to reduce local inflammation. They have decreased antigen presentation capability, limited oxidant production, and increased production of anti-inflammatory cytokines, which helps prevent excessive tissue damage. The balance between M1/M2 macrophages in an organ during inflammation or injury can determine its fate. When this balance is disrupted, macrophages can contribute to tissue damage and necrosis. STB is a chronic infectious disease, and local immune status is a significant factor in Mycobacterium tuberculosis survival and tissue destruction. M1 macrophages, which produce high levels of inflammatory cytokines and proteolytic enzymes in the context of chronic inflammation, can contribute to spine malformation and SCI. Previous studies conducted by our team revealed a significant increase in M1 macrophages in STB patients^47^. Therefore, it is reasonable to consider that monocytes and macrophages may be significant factors contributing to SCI in patients with STB.

Despite numerous key factors being associated with SCI in patients with spinal tuberculosis, no predictive model has been developed. ML-based predictive models are gaining popularity due to their precision and are increasingly applied in spinal diseases treatment^48^. In this study, ten meticulously chosen features were employed to construct predictive models. To establish the reliability of our findings, ten machine learning algorithms were utilized in the creation of these models. Our comprehensive evaluation of the results, considering measures such as the AUC, precision-recall curves, decision curve analysis, and calibration curves, indicated that the random forest (RF) model outperformed the other nine models. The learning curve also indicated that the RF model exhibited effective performance, underscoring the clinical value of ML models in predicting spinal cord injury in patients with spinal tuberculosis. Furthermore, additional clinical data were collected to validate the model externally, ensuring its generalizability and reproducibility, which are essential for translating our results into clinical practice.

In recent years, the introduction of advanced machine learning models, particularly black box models, has significantly propelled the state-of-the-art in various domains^49^. Models like deep neural networks and ensemble methods have demonstrated unparalleled performance in tackling complex tasks. However, their widespread adoption has given rise to a critical concern—the inherent opacity in comprehending their decision-making processes. This opacity results from the intricate interplay of features and parameters, posing a challenge for human comprehension. Not only does this opacity hinder interpretability, but it also raises crucial issues related to trust, accountability, and ethical considerations. In response to these challenges, there has been a growing emphasis on the development and adoption of explainable artificial intelligence (XAI) methods, such as Local Interpretable Model-agnostic Explanations (LIME) or SHapley Additive exPlanations (SHAP)^50^. In our study, to provide a more comprehensive understanding of the predictive model and address the opacity of black-box models, we employed the DALEX R package to assess feature importance. This analysis revealed key indicators associated with spinal tuberculosis. Additionally, we utilized SHAP to interpret single-sample predictions of the model, aiming to enhance transparency and interpretability in our findings.

While the results show promise, it is important to acknowledge several limitations in this study. First, despite being a multicenter study, the sample size was relatively small, which could introduce bias into several of the results. One potentially effective strategy is the application of generative methods for data augmentation. By employing techniques such as rotation, scaling, and flipping, we aim to artificially create additional training samples, thereby expanding the dataset. This approach has been widely utilized in the literature, as evidenced by the work mentioned in reference^51,52^, while other approaches include transfer learning, active learning, adversarial training, and so on. Second, to enhance the accuracy and performance of the model, it would have been beneficial to include more favorable clinical indicators related to the prognosis of spinal tuberculosis, such as radiomic features. Third, even though prospective studies were conducted to enhance the reliability and generalizability of our findings, data collection uncertainties in prospective cohorts from different regions may lead to unavoidable bias. Finally, the potential molecular mechanisms underlying the key factors for determining the prognosis of spinal tuberculosis patients have not been elucidated.

## Conclusions

In summary, this study successfully developed a valuable predictive model for spinal cord injury in patients with spinal tuberculosis. This model was created using a combination of multiple machine-learning algorithms and data from multiple clinical centers. Furthermore, we established a personalized risk assessment tool for spinal cord injury in spinal tuberculosis patients. Finally, we deployed the model on the web. Notably, monocytes may play a key role in the development of spinal cord injury in these patients, according to the variable importance ranking. This research offers an efficient and rapid approach for frontline clinicians and patients to predict the risk of spinal cord injury in patients with spinal tuberculosis and provides valuable guidance for clinical decision-making.

### Supplementary Information

Supplementary Table 1.

Supplementary Table 2.

## Data Availability

The original data utilized herein in the study are included in the article. Further inquiries can be directed to the corresponding author.

## References

[1] Chakaya J, Khan M, Ntoumi F, Aklillu E, Fatima R, Mwaba P (2021). Global tuberculosis report 2020—Reflections on the global TB burden, treatment and prevention efforts. Int. J. Infect. Dis..

[2] Furin J, Cox H, Pai M (2019). Tuberculosis. Lancet.

[3] Dunn RN, Ben HM (2018). Spinal tuberculosis: Review of current management. Bone Jt. J..

[4] Jain AK, Rajasekaran S, Jaggi KR, Myneedu VP (2020). Tuberculosis of the Spine. J. Bone Jt. Surg. Am..

[5] Garcia-Rodriguez JF, Alvarez-Diaz H, Lorenzo-Garcia MV, Marino-Callejo A, Fernandez-Rial A, Sesma-Sanchez P (2011). Extrapulmonary tuberculosis: Epidemiology and risk factors. Enferm. Infecc. Microbiol. Clin..

[6] Khanna K, Sabharwal S (2019). Spinal tuberculosis: A comprehensive review for the modern spine surgeon. Spine J..

[7] Kim J-H, Kim ES, Jun K-I, Hg Jung, Bang JH, Choe PG (2018). Delayed diagnosis of extrapulmonary tuberculosis presenting as fever of unknown origin in an intermediate-burden country. BMC Infect. Dis..

[8] Gilpin C, Korobitsyn A, Migliori GB, Raviglione MC, Weyer K (2018). The World Health Organization standards for tuberculosis care and management. Eur. Respir. J..

[9] Margraf JT (2023). Science-driven atomistic machine learning. Angew. Chem. Int. Ed. Engl..

[10] Srinivas S, Young AJ (2023). Machine learning and artificial intelligence in surgical research. Surg. Clin. N. Am..

[11] Ota R, Yamashita F (2022). Application of machine learning techniques to the analysis and prediction of drug pharmacokinetics. J. Control Release.

[12] Guo T, Li X (2023). Machine learning for predicting phenotype from genotype and environment. Curr. Opin. Biotechnol..

[13] Mondal PP, Galodha A, Verma VK, Singh V, Show PL, Awasthi MK (2023). Review on machine learning-based bioprocess optimization, monitoring, and control systems. Bioresour. Technol..

[14] Duan S, Dong W, Hua Y, Zheng Y, Ren Z, Cao G (2023). Accurate differentiation of spinal tuberculosis and spinal metastases using MR-based deep learning algorithms. Infect. Drug Resist..

[15] Li Z, Wu F, Hong F, Gai X, Cao W, Zhang Z (2022). Computer-aided diagnosis of spinal tuberculosis from CT images based on deep learning with multimodal feature fusion. Front. Microbiol..

[16] Zhou C, Liang T, Jiang J, Chen J, Chen T, Huang S (2023). MMP9 and STAT1 are biomarkers of the change in immune infiltration after anti-tuberculosis therapy, and the immune status can identify patients with spinal tuberculosis. Int. Immunopharmacol..

[17] Wu S, Liang T, Jiang J, Zhu J, Chen T, Zhou C (2023). Proteomic analysis to identification of hypoxia related markers in spinal tuberculosis: A study based on weighted gene co-expression network analysis and machine learning. BMC Med. Genom..

[18] Chen L, Liu C, Liang T, Ye Z, Huang S, Chen J (2022). Mechanism of COVID-19-related proteins in spinal tuberculosis: Immune dysregulation. Front. Immunol..

[19] Borislavov L, Nedyalkova M, Tadjer A, Aydemir O, Romanova J (2023). Machine learning-based screening for potential singlet fission chromophores: The challenge of imbalanced data sets. J. Phys. Chem. Lett..

[20] Jiang X, Xu C (2022). Deep learning and machine learning with grid search to predict later occurrence of breast cancer metastasis using clinical data. J. Clin. Med..

[21] He J, Wang B, Tao J, Liu Q, Peng M, Xiong S (2023). Accurate classification of pulmonary nodules by a combined model of clinical, imaging, and cell-free DNA methylation biomarkers: A model development and external validation study. Lancet Digit Health.

[22] Obuchowski NA, Bullen JA (2018). Receiver operating characteristic (ROC) curves: Review of methods with applications in diagnostic medicine. Phys. Med. Biol..

[23] Li W, Guo Q (2021). Plotting receiver operating characteristic and precision-recall curves from presence and background data. Ecol. Evol..

[24] Vickers AJ, Elkin EB (2006). Decision curve analysis: A novel method for evaluating prediction models. Med. Decis. Mak..

[25] Fenlon C, O'Grady L, Doherty ML, Dunnion J (2018). A discussion of calibration techniques for evaluating binary and categorical predictive models. Prev. Vet. Med..

[26] Scodari BT, Chacko S, Matsumura R, Jacobson NC (2023). Using machine learning to forecast symptom changes among subclinical depression patients receiving stepped care or usual care. J. Affect. Disord..

[27] Li J, Liu S, Hu Y, Zhu L, Mao Y, Liu J (2022). Predicting mortality in intensive care unit patients with heart failure using an interpretable machine learning model: Retrospective cohort study. J. Med. Internet Res..

[28] Belkin M, Hsu D, Ma S, Mandal S (2019). Reconciling modern machine-learning practice and the classical bias-variance trade-off. Proc. Natl. Acad. Sci. U. S. A..

[29] Peghin M, Rodriguez-Pardo D, Sanchez-Montalva A, Pellise F, Rivas A, Tortola T (2017). The changing epidemiology of spinal tuberculosis: the influence of international immigration in Catalonia, 1993–2014. Epidemiol. Infect..

[30] Chen SH, Lin WC, Lee CH, Chou WY (2008). Spontaneous infective spondylitis and mycotic aneurysm: Incidence, risk factors, outcome and management experience. Eur. Spine J..

[31] Xu G, Xue J, Jiang J, Liang T, Yao Y, Liao S (2021). Proteomic analysis reveals critical molecular mechanisms involved in the macrophage anti-spinal tuberculosis process. Tuberculosis (Edinb.).

[32] Sun Z, Pang X, Wang X, Zeng H (2023). Differential expression analysis of miRNAs in macrophage-derived exosomes in the tuberculosis-infected bone microenvironment. Front. Microbiol..

[33] Yao Y, Wu S, Liu C, Zhou C, Zhu J, Chen T (2023). Identification of spinal tuberculosis subphenotypes using routine clinical data: A study based on unsupervised machine learning. Ann. Med..

[34] Yang L, Gao C, Li F, Yang L, Chen J, Guo S (2021). Monocyte-to-lymphocyte ratio is associated with 28-day mortality in patients with acute respiratory distress syndrome: A retrospective study. J. Intensive Care.

[35] Muller BL, Ramalho DM, Santos PF, Mesquita ED, Kritski AL, Oliveira MM (2013). Inflammatory and immunogenetic markers in correlation with pulmonary tuberculosis. J. Bras. Pneumol..

[36] Kim JH, Ahn JY, Jeong SJ, Ku NS, Choi JY, Kim YK (2019). Prognostic factors for unfavourable outcomes of patients with spinal tuberculosis in a country with an intermediate tuberculosis burden: A multicentre cohort study. Bone Jt. J..

[37] Tang L, Fu CG, Zhou ZY, Jia SY, Liu ZQ, Xiao YX (2022). Clinical features and outcomes of spinal tuberculosis in central China. Infect. Drug Resist..

[38] Huang Y, Wu R, Xia Q, Liu L, Feng G (2023). Prognostic values of geriatric nutrition risk index on elderly patients after spinal tuberculosis surgery. Front. Nutr..

[39] Chen L, Liu C, Liang T, Ye Z, Huang S, Chen J (2022). Monocyte-to-lymphocyte ratio was an independent factor of the severity of spinal tuberculosis. Oxid. Med. Cell Longev..

[40] Luo M, Zou X, Zeng Q, Wu Y, Yang H, Qin L (2023). Monocyte at diagnosis as a prognosis biomarker in tuberculosis patients with anemia. Front. Med. (Lausanne).

[41] Nonaka M, Matsuyama M, Sakai C, Matsumura S, Arai N, Nakajima M (2023). Risk factors for clinical progression in patients with pulmonary Mycobacterium avium complex disease without culture-positive sputum: A single-center, retrospective study. Eur. J. Med. Res..

[42] Trovato FM, Zia R, Artru F, Mujib S, Jerome E, Cavazza A (2023). Lysophosphatidylcholines modulate immunoregulatory checkpoints in peripheral monocytes and are associated with mortality in people with acute liver failure. J. Hepatol..

[43] Orchanian SB, Lodoen MB (2023). Monocytes as primary defenders against *Toxoplasma*
*gondii* infection. Trends Parasitol..

[44] Hou P, Fang J, Liu Z, Shi Y, Agostini M, Bernassola F (2023). Macrophage polarization and metabolism in atherosclerosis. Cell Death Dis..

[45] Meidaninikjeh S, Sabouni N, Marzouni HZ, Bengar S, Khalili A, Jafari R (2021). Monocytes and macrophages in COVID-19: Friends and foes. Life Sci..

[46] Sia JK, Rengarajan J (2019). Immunology of *Mycobacterium*
*tuberculosis* infections. Microbiol. Spectr..

[47] Liang T, Chen J, Xu G, Zhang Z, Xue J, Zeng H (2022). STAT1 and CXCL10 involve in M1 macrophage polarization that may affect osteolysis and bone remodeling in extrapulmonary tuberculosis. Gene.

[48] Galbusera F, Casaroli G, Bassani T (2019). Artificial intelligence and machine learning in spine research. JOR Spine.

[49] Garrett BL, Rudin C (2023). Interpretable algorithmic forensics. Proc. Natl. Acad. Sci. U. S. A..

[50] van der Velden BHM, Kuijf HJ, Gilhuijs KGA, Viergever MA (2022). Explainable artificial intelligence (XAI) in deep learning-based medical image analysis. Med. Image Anal..

[51] Zhang C, Bao N, Sun H, Li H, Li J, Qian W (2022). A deep learning image data augmentation method for single tumor segmentation. Front. Oncol..

[52] Cheung TH, Yeung DY (2023). A survey of automated data augmentation for image classification: Learning to compose, mix, and generate. IEEE Trans. Neural Netw. Learn. Syst..

