# Supplementary material for: Prediction model for spinal cord injury in spinal tuberculosis patients using multiple machine learning algorithms: a multicentric study
Source: Sci Rep. 2024 Apr 2;14:7691. doi: 10.1038/s41598-024-56711-0 (PMC10987632; doi:10.1038/s41598-024-56711-0)
Supplement: Supplementary file 1 — Supplementary Table 1. [file 41598_2024_56711_MOESM1_ESM.docx]

Table 1 Baseline characteristics of STB patients with and without SCI in validation set

| Characteristics | No.(%) | | | p |
| --- | --- | --- | --- | --- |
|  | Total | SCI | No-SCI |  |
| Age,years, Mean±SD | 50.7±17.3 | 53.6±17.2 | 46.9±17 | 0.113 |
| BMI, median(IQR) | 20.2(18.6-22) | 19.8(18.3-21.9) | 20.5(19.3-22.3) | 0.446 |
| Sex, n(%) |  |  |  | 0.589 |
| Male | 56(67.5%) | 36(43.4%) | 20(24.1%) |  |
| Female | 27(32.5%) | 11(13.3%) | 16(19.2%) |  |
| Diabetes, n(%) |  |  |  | 0.461 |
| Yes | 7(8.4%) | 3(3.6%) | 4(4.8%) |  |
| No | 76(91.6%) | 44(53%) | 32(38.6%) |  |
| Hypertension, n(%) |  |  |  | 0.626 |
| Yes | 24(28.9%) | 15(18.1%) | 9(10.8%) |  |
| No | 59(71.1%) | 32(38.6%) | 27(32.5%) |  |
| CRP, median(IQR) | 12.3(6.28-22.6) | 14.3(6.94-22.7) | 10.3(4.1-22.72) | 0.291 |
| WBC*10^9/L, median(IQR) | 7.3(5.96-8.5) | 7.65(6.37-9.07) | 6.42(5.62-8.17) | 0.028 |
| NEU*10^9/L, median(IQR) | 4.45(3.6-6.04) | 5(4-6.8) | 3.9(3.44-5.36) | 0.011 |
| LYM*10^9/L, median(IQR) | 1.49(1.15-1.9) | 1.4(1-1.93) | 1.63(1.28-1.78) | 0.168 |
| MONO*10^9/L, median(IQR) | 0.62(0.51-0.79) | 0.69(0.52-0.84) | 0.54(0.49-0.71) | 0.022 |
| HGB g/L, median(IQR) | 123(110-135) | 123(106-131) | 124(111-135) | 0.779 |
| PLT *10^9/L, median(IQR) | 279(236-350) | 290(239-362.9) | 262(236.6-334) | 0.363 |
| ESR, median(IQR) | 35(17-54.5) | 36(18-56) | 34.5(15.5-54) | 0.44 |
| ALB g/L, median(IQR) | 37.6(34.8-40.7) | 37.2(34.7-40.6) | 38.1(35-40.8) | 0.448 |
| TP g/L, median(IQR) | 68.8(65.7-74.6) | 68.6(65-73.3) | 69.3(66-74.9) | 0.55 |
| AST u/L, median(IQR) | 21(17.5-27) | 22(18.5-27) | 19(15-22.8) | 0.041 |
| ALT u /L, median(IQR) | 17(12-25.5) | 17(11.5-31) | 17(12-21.8) | 0.499 |
| Ure u/L, median(IQR) | 4.39(3.22-5.5) | 4.12(3.26-5.58) | 4.46(3.17-5.41) | 0.673 |
| Scr u/L, Mean±SD | 67±15.1 | 67±14.2 | 67±16.5 | 0.898 |
| UA u/L, median(IQR) | 355(264-496) | 368(269-600.5) | 338(261-436) | 0.102 |
