# Supplementary material for: Prediction model for spinal cord injury in spinal tuberculosis patients using multiple machine learning algorithms: a multicentric study
Source: Sci Rep. 2024 Apr 2;14:7691. doi: 10.1038/s41598-024-56711-0 (PMC10987632; doi:10.1038/s41598-024-56711-0)
Supplement: Supplementary file 2 — Supplementary Table 2. [file 41598_2024_56711_MOESM2_ESM.docx]

Table 2 Baseline characteristics of STB patients with and without SCI in testing set

| Characteristics | No.(%) | | | p |
| --- | --- | --- | --- | --- |
|  | Total | SCI | No-SCI |  |
| Age,years, median(IQR) | 56.5(48.8-64) | 58(50-65) | 55.5(45.5-58.5) | 0.1689 |
| BMI, median(IQR) | 19.4(17.9-20.9) | 19.4(17.9-20.5) | 20.1(17.9-22) | 0.4122 |
| Sex, n(%) |  |  |  | 0.927 |
| Male | 42(52.5%) | 25(31.3%) | 17(21.2%) |  |
| Female | 38(47.5%) | 23(28.8%) | 15(18.8%) |  |
| Diabetes, n(%) |  |  |  | 0.079 |
| Yes | 15(18.8%) | 12(15%) | 3(3.8%) |  |
| No | 65(81.2%) | 36(45%) | 29(36.2%) |  |
| Hypertension, n(%) |  |  |  | 0.021 |
| Yes | 27(33.6%) | 21(26.3%) | 6(7.3%) |  |
| No | 53(66.4%) | 27(33.9%) | 26(32.5%) |  |
| CRP, median(IQR) | 24(6.2-62.7) | 31.2(11.7-82.2) | 8.71(4.22-39.5) | 0.0071 |
| WBC*10^9/L, median(IQR) | 5.32(5.01-10.3) | 6.38(4.73-9.16) | 6.5(5.08-8.2) | 0.5959 |
| NEU*10^9/L, median(IQR) | 4.25(2.8-5.49) | 4.3 (2.64-7.28) | 4.24(3.08-5.14) | 0.6165 |
| LYM*10^9/L, median(IQR) | 1.21(0.85-2.04) | 1.13(0.83-1.36) | 1.69(0.94-2.19) | 0.1094 |
| MONO*10^9/L, median(IQR) | 0.65(0.48-0.82) | 0.68(0.55-0.95) | 0.58(0.43-0.75) | 0.0181 |
| HGB g/L, median(IQR) | 114(102-128) | 111(98-118) | 127(108-135) | 0.0047 |
| PLT *10^9/L, median(IQR) | 308(244-407) | 362(292-427) | 251(219-319.3) | 0.0020 |
| ESR, median(IQR) | 42(25-60) | 52.5(34-66.5) | 30.5(17.8-44.8) | 0.0006 |
| ALB g/L, median(IQR) | 36.3(33.8-40.2) | 35(32.2-39.15) | 37.4(35.6-40.7) | 0.042 |
| TP g/L, median(IQR) | 69.4(65.3-72.7) | 69.9(65.6-73.6) | 69(64.3-71.4) | 0.2029 |
| AST u/L, median(IQR) | 23(17-33.2) | 25(19-37.81) | 20.5(16-28.3) | 0.082 |
| ALT u /L, median(IQR) | 18(11-35) | 16.5(10-35.5) | 21.5(14-32) | 0.1879 |
| Ure u/L, median(IQR) | 4.54(3.78-5.54) | 4.7(3.76-6.18) | 4.38(3.81-5.33) | 0.6836 |
| Scr u/L, Mean±SD | 68.5(52-82.2) | 60(50-79.75) | 70.5(58-83.3) | 0.1630 |
| UA u/L, median(IQR) | 391(276-544) | 386(254-561) | 398(311-524) | 0.8713 |
